# Supplementary material for: Low-Carbon Travel Motivation and Constraint: Scales Development and Validation
Source: Int J Environ Res Public Health. 2022 Apr 22;19(9):5123. doi: 10.3390/ijerph19095123 (PMC9105957; doi:10.3390/ijerph19095123)
Supplement: Supplementary file 1 [file ijerph-19-05123-s001.zip › ijerph-1661699-supplementary.pdf]

File S1. The open-ended interview questions.

1. Based on your past tourism experience and your understanding of low-carbon tourism, what personal internal needs will you consider to engage in low-carbon tourism, or do energy-saving and carbon reducing tourism activities in tourism (such as reducing personal carbon footprint and pursuing health)? Please specify the event content.

2. Based on your past travel experience and your understanding of low-carbon tourism, what characteristics / factors of low-carbon tourism process will attract you to engage in low-carbon tourism (such as energy-saving and carbon reducing rooms and facilities, discount incentives for low-carbon consumption)? Please specify the event content.

3. Based on your past travel experience and your understanding of low-carbon tourism, what personal factors will you not engage in low-carbon tourism or do energy-saving and carbon reducing tourism in tourism (e.g., lack of time, lack of funds and low-carbon information)? Please specify the event content.

4. Based on your past travel experience and your understanding of low-carbon tourism, what other factors (family, friends, peers, government, enterprises, idols... And other reference groups) will you not engage in low-carbon tourism, or do energy-saving and carbon reduction tourism in tourism (for example, without peer friends, the responsibility of enterprise energy-saving and carbon reduction is greater than personal responsibility)? Please specify the event content.

5. Based on your past travel experience and your understanding of low-carbon tourism, what are the characteristics of low-carbon tourism that you will not engage in low-carbon tourism, or do energy-saving and carbon reducing tourism activities in tourism (for example, inconvenient low-carbon activities and uncomfortable low-carbon tourism methods)? Please specify the event content.
